# Manometric and demographic predictors of incomplete bolus transit in patients diagnosed with ineffective esophageal motility

**DOI:** 10.1186/1753-6561-9-S7-A14

**Published:** 2015-10-27

**Authors:** Sarah Pradhan, Michelle Buresia, Milli Gupta, Michael Curley, Lynn Wilsack, Christopher N Andrews

**Affiliations:** 1 Royal College of Surgeons in Ireland, Dublin, Ireland; 2 University of Calgary, Calgary, Canada

## Background

Ineffective esophageal motility (IEM), or frequent failed peristalsis in the Chicago Classification, is a common motility abnormality describing a non-specific manometric pattern of peristaltic failure. With the addition of multichannel intraluminal impedance technology to high resolution esophageal manometry (HRM), evaluation of bolus transit simultaneously with esophageal contraction is possible. However, manometric predictors of incomplete bolus transit (IBT) in the setting of IEM have not been fully characterized.

## Methods

REB-approved retrospective chart review of patients diagnosed with IEM at a regional gut motility centre. All subjects were clinically assessed prior to HRM with a detailed history containing the patient's primary complaints, other pertinent symptoms and demographic information. HRM with impedance studies (Given Imaging, Inc) were performed by standard protocol. Summary (averaged) data from each patient was compared. To examine which manometric variables best predicted the percentage of swallows with incomplete bolus transit for any given patient, a multiple linear regression was performed using selected variables, and adjusted for age and gender (SPSS 21). All tests were two-sided and significance was set at the 95% level.

## Results

230 patients (130 female; mean age 52 yr; range 18-82 yr) with a manometric diagnosis of IEM were included. The primary complaints of patients included dysphagia (33.9%), heartburn/reflux (33.0%), chest pain (11.7%), other (10.9%), and cough (8.7%). Many patients had more than one symptom; however there were no significant differences in baseline characteristics across primary symptom groups (data not shown). The multiple regression model fit was highly significant (F=10.14, 9 df, p<0.001) with an adjusted r square of 0.373. In the model, decreasing proportions of peristaltic contractions correlated significantly with decreased bolus transit (ie higher percentage of incomplete bolus transit). Similarly, higher intrabolus pressure correlated significantly with higher percentages of incomplete bolus transit. The remainder of the manometric variables were not significantly associated with IBT. Gender was not significant but increasing age was associated with less IBT.

## Conclusions

As expected, peristaltic contractions predict adequate bolus transit in aggregate data, while increased intrabolus pressure predicted incomplete bolus transit. Further questions being evaluated in this study sample include effects of medications and connective tissue disorders on HRM and bolus transit variables in the setting of IEM.

